# Computation of the distribution of model accuracy statistics in machine learning: Comparison between analytically derived distributions and simulation‐based methods

**DOI:** 10.1002/hsr2.1214

**Published:** 2023-04-20

**Authors:** Alexander A. Huang, Samuel Y. Huang

**Affiliations:** ^1^ Northwestern University Feinberg School of Medicine Northwestern University Chicago Illinois USA; ^2^ Virginia Commonwealth School of Medicine Virginia Commonwealth University Richmond Virginia USA

**Keywords:** Anderson–Darling, bootstrap, Gaussian distribution, normal distribution, simulation, sufficient statistics, variance calculations, Whitney–Mann

## Abstract

**Background and Aims:**

All fields have seen an increase in machine‐learning techniques. To accurately evaluate the efficacy of novel modeling methods, it is necessary to conduct a critical evaluation of the utilized model metrics, such as sensitivity, specificity, and area under the receiver operator characteristic curve (AUROC). For commonly used model metrics, we proposed the use of analytically derived distributions (ADDs) and compared it with simulation‐based approaches.

**Methods:**

A retrospective cohort study was conducted using the England National Health Services Heart Disease Prediction Cohort. Four machine learning models (XGBoost, Random Forest, Artificial Neural Network, and Adaptive Boost) were used. The distribution of the model metrics and covariate gain statistics were empirically derived using boot‐strap simulation (*N* = 10,000). The ADDs were created from analytic formulas from the covariates to describe the distribution of the model metrics and compared with those of bootstrap simulation.

**Results:**

XGBoost had the most optimal model having the highest AUROC and the highest aggregate score considering six other model metrics. Based on the Anderson–Darling test, the distribution of the model metrics created from bootstrap did not significantly deviate from a normal distribution. The variance created from the ADD led to smaller SDs than those derived from bootstrap simulation, whereas the rest of the distribution remained not statistically significantly different.

**Conclusions:**

ADD allows for cross study comparison of model metrics, which is usually done with bootstrapping that rely on simulations, which cannot be replicated by the reader.

## 1 INTRODUCTION

All fields, from computer science to medicine, have significantly increased their use of machine learning algorithms.^1^, ^2^, ^3^, ^4^, ^5^, ^6^, ^7^ These algorithms are unique in that they can predict data without explicit instructions from the modeler.^1^, ^5^, ^8^, ^9^ XGBoost and Random Forest, two well‐known machine learning algorithms, have been shown to be significantly more accurate than linear and logistic regression.^4^, ^10^, ^11^ However, in the case of machine learning (ML) algorithms, decreased interpretability comes at the cost of increased predictive accuracy, and ML algorithms are frequently referred to as “black boxes” because they cannot be understood.^1^, ^5^, ^7^, ^8^, ^9^, ^10^, ^11^ When evaluating these methods, researchers heavily rely on model metrics like area under the receiver operator characteristic curve (AUROC), sensitivity, specificity, and accuracy.^1^, ^2^, ^4^

Bootstrap is commonly the method of choice to calculate the distribution of these model metrics.^2^, ^12^, ^13^, ^14^, ^15^ As a simulation‐based method, a reader cannot rerun the simulation the same way they can back‐calculate from a computed *t* test.^16^, ^17^, ^18^, ^19^, ^20^ It was necessary to develop analytically derived distributions (ADDs) to accurately summarize the distribution of these model metrics the same way a Gaussian distribution summarizes the mean and SD for accurate comparison of models within and between studies.

This aim of the study is to compare ADD created from formulas in the statistical literature from those derived from bootstrapping the distribution of model statistics.

## 2 METHODS

The Heart Disease Prediction cohort from the England National Health Services database was used in this retrospective cohort study.^21^, ^22^, ^23^ All methods in this research were carried out in accordance with guidelines detailed by the Data Alliance Partnership Board‐approved national information standards and data collections for use in health and adult social care. This code was written using R version 4.2.2. We used the following packages readxl and foreign for reading in the data set, MLDataR for the data set, dplyr and ggplot2 for datavisualization, xgboost, farff, tibble, lspline, and pROC for model creation.

### 2.1 Model metrics of interest

The model metrics were selected due to their prevalence in the literature and included the AUROC, sensitivity, specificity, positive predictive value (PPV), negative predictive value (NPV), F1, accuracy, and balanced accuracy. Metrics of feature importance assessed in this study included the gain, cover, and frequency.

### 2.2 Independent variables

Demographic covariates included age and sex. Clinical covariates included resting blood pressure, fasting blood sugar, cholesterol, resting electrocardiogram (ECG), presence of angina, and maximum heart rate.

### 2.3 Dependent variable

The dependent variable of interest was a clinician's diagnosis of heart disease.

### 2.4 Model construction and statistical analysis

Descriptive statistics for all patients and then patients stratified by heart disease were computed for all covariates and compared using *χ* ^2^ tests for categorical variables and *t* tests for continuous variables. Machine learning methods including XGBoost, Random Forest, Artificial Neural Network, and Adaptive Boosting were implemented on the data set.

### 2.5 Bootstrap simulation and ADD compared via distribution of model metrics

#### 2.5.1 Distribution evaluation

The distribution of each of the statistics was evaluated through comparison of summary statistics (minimum, 5th percentile, 25th percentile, 50th percentile, 75th percentile, 95th percentile, maximum, mean, SD) and the Anderson–Darling test for normality.

### 2.6 Bootstrap simulation

A train‐test set (70:30) was used within all machine‐learning models in this study. Bootstrap simulation (*N* = 10,000) simulations were carried out by permuting the train‐test sets before training.

### 2.7 Calculation of variance with analytical formulas to create the ADD

#### 2.7.1 AUROC

The AUROC $=\frac{U}{n^{2}}$ where *U* has the Mann–Whitney distribution: $U=\sum_{i=1}^{n}\sum_{j=1}^{m}F(X_{i},Y_{j})$, where $F\;(X_{i},Y_{j})=\left\{\begin{array}{ll} 1 & X>Y\\ 1 & X=Y\\ 0 & X<Y \end{array}\right.$, and $X_{1},\ldots ,X_{n}\&\;Y_{1},\ldots Y_{m}$ are individually identically distributed. As *U* has the Mann–Whitney distribution, we observe that the variance of the *U* distribution is $\sigma_{U}^{2}=\frac{n^{2}(2n+1)}{12}$. Thus, as AUROC $=\frac{U}{n^{2}},\sigma_{\mathrm{AUROC}}^{2}=\frac{\sigma_{U}^{2}}{{\left(n^{2}\right)}^{2}}=\frac{\sigma_{U}^{2}}{n^{4}}=\frac{n^{2}(2n+1)}{12n^{4}}=\frac{\left(2n+1\right)}{12n^{2}}.$ As the Mann–Whitney distribution is asymptomatically convergent on the Gaussian distribution at large sample sizes, the mean and SD are sufficient statistics. We further observe that for large *n*, the variance formula for the AUROC can be approximated as: $\sigma_{\mathrm{AUROC}}^{2}=\frac{\left(2n+1\right)}{12n^{2}}\to \frac{\left(2n\right)}{12n^{2}}=\frac{1}{6n}$. Furthermore, another more nuanced measurement for the variability can also be dependent on the value of AUROC itself and approximating it as a proportion yields similar approximation to the Mann–Whitney distribution for when the values of AUROC are between 0.7 and 0.9. Thus, another similarly correct analytic approximation of the AUROC is $\sigma_{\mathrm{AUROC}}^{2}=\frac{\left(\mathrm{AUROC})(1-\mathrm{AUROC}\right)}{n}$.

### 2.8 Model metrics that can be evaluated as proportions

Multiple literature sources have treated accuracy, F1, sensitivity, specificity, PPV, and NPV as similar to proportions. Thus, we make the assumption from treating these statistics as proportions that their variance follows from the analytic formula: $\sigma_{p}^{2}=\frac{\left(p)(1-p\right)}{n}\;$, where *p* is the proportion. Thus, the SDs for the model metrics are: accuracy: $\sigma_{\mathrm{Accuracy}}^{2}=\frac{\left(\mathrm{Accuracy})(1-\mathrm{Accuracy}\right)}{n}$, F1: $\sigma_{F1}^{2}=\frac{\left(F1)(1-F1\right)}{n}$, Sensitivity: $\sigma_{\mathrm{Sensitivity}}^{2}=\frac{\left(\mathrm{Sensitivity})(1-\mathrm{Sensitivity}\right)}{n}$, Specificity: $\sigma_{\mathrm{Specificity}}^{2}=\frac{\left(\mathrm{Specificity})(1-\mathrm{Specificity}\right)}{n}$, PPV: $\sigma_{\mathrm{PPV}}^{2}=\frac{\left(\mathrm{PPV})(1-\mathrm{PPV}\right)}{n}$, and NPV: $\sigma_{\mathrm{NPV}}^{2}=\frac{\left(\mathrm{NPV})(1-\mathrm{NPV}\right)}{n}$.

## 3 RESULTS

Table 1a shows the model metrics of the four machine learning models calculated for accuracy, F1, sensitivity, specificity, PPV, NPV, and AUROC utilizing the bootstrap method. Table 1b shows the model metrics of the four machine learning models calculated for accuracy, F1, sensitivity, specificity, PPV, NPV, and AUROC utilizing ADD. The distributions of the model metrics for the bootstrap method and ADD are approximately similar.

**Table 1a:** Summary of model metrics.

	Metrics	Minimum	5th Percentile	25th Percentile	Median	75th Percentile	95th Percentile	Maximum	Mean	SD	Range
**XGBoost**	Accuracy	0.684	0.741	0.766	0.790	0.806	0.836	0.898	0.789	0.026	0.210
	F1	0.686	0.750	0.774	0.784	0.810	0.835	0.897	0.787	0.031	0.204
	Sensitivity	0.680	0.757	0.790	0.806	0.821	0.853	0.901	0.802	0.026	0.224
	Specificity	0.592	0.708	0.749	0.786	0.815	0.852	0.947	0.789	0.037	0.348
	PPV	0.680	0.761	0.787	0.818	0.847	0.884	0.958	0.818	0.035	0.273
	NPV	0.567	0.676	0.722	0.753	0.785	0.829	0.930	0.761	0.046	0.354
	AUROC	0.772	0.831	0.856	0.867	0.884	0.903	0.948	0.868	0.025	0.171
**Random Forest**	Accuracy	0.675	0.729	0.771	0.778	0.801	0.812	0.892	0.784	0.027	0.224
	F1	0.687	0.740	0.771	0.776	0.809	0.816	0.884	0.785	0.030	0.201
	Sensitivity	0.665	0.745	0.782	0.799	0.804	0.847	0.895	0.793	0.024	0.229
	Specificity	0.584	0.709	0.748	0.784	0.803	0.845	0.927	0.771	0.041	0.340
	PPV	0.676	0.740	0.779	0.813	0.846	0.857	0.948	0.810	0.045	0.270
	NPV	0.555	0.661	0.720	0.736	0.772	0.826	0.908	0.750	0.045	0.359
	AUROC	0.757	0.824	0.842	0.860	0.887	0.900	0.928	0.857	0.022	0.175
**Artificial Neural Network**	Accuracy	0.689	0.736	0.761	0.786	0.805	0.830	0.877	0.781	0.021	0.194
	F1	0.677	0.732	0.750	0.783	0.790	0.818	0.888	0.774	0.027	0.211
	Sensitivity	0.672	0.749	0.779	0.794	0.802	0.834	0.884	0.796	0.021	0.216
	Specificity	0.591	0.707	0.749	0.768	0.799	0.835	0.928	0.768	0.035	0.327
	PPV	0.659	0.748	0.780	0.809	0.835	0.859	0.940	0.808	0.029	0.274
	NPV	0.550	0.665	0.718	0.752	0.772	0.817	0.912	0.749	0.047	0.361
	AUROC	0.751	0.821	0.839	0.866	0.882	0.891	0.949	0.847	0.027	0.192
**Adaptive Boosting**	Accuracy	0.683	0.731	0.761	0.790	0.793	0.821	0.885	0.775	0.023	0.199
	F1	0.674	0.739	0.760	0.774	0.801	0.828	0.890	0.775	0.029	0.224
	Sensitivity	0.671	0.753	0.783	0.811	0.809	0.839	0.889	0.797	0.019	0.216
	Specificity	0.585	0.694	0.746	0.777	0.803	0.853	0.941	0.772	0.045	0.354
	PPV	0.676	0.742	0.772	0.805	0.843	0.861	0.951	0.817	0.045	0.277
	NPV	0.567	0.664	0.717	0.751	0.784	0.825	0.929	0.750	0.047	0.358
	AUROC	0.755	0.815	0.840	0.860	0.865	0.894	0.929	0.862	0.025	0.175

*Note*: Summary of model metrics within the test set for each of the four machine learning techniques (XGBoost, Random Forest, Artificial Neural Network, and Adaptive Boosting) based upon bootstrap simulation.

Abbreviations: AUROC, area under the receiver operator characteristic curve; NPV, negative predictive value; PPV, positive predictive value.

**Table 1b:** Summary of model metrics for each of the four machine learning techniques.

	**Metrics**	**Minimum**	**5th Percentile**	**25th Percentile**	**Median**	**75th Percentile**	**95th Percentile**	**Maximum**	**Mean**	**SD**	**Range**
**XGBoost**	Accuracy	0.684	0.751	0.773	0.789	0.805	0.828	0.898	0.789	0.024	0.215
	F1	0.686	0.748	0.771	0.787	0.803	0.826	0.897	0.787	0.024	0.210
	Sensitivity	0.680	0.764	0.787	0.802	0.818	0.840	0.901	0.802	0.023	0.222
	Specificity	0.592	0.750	0.773	0.789	0.805	0.827	0.947	0.789	0.024	0.354
	PPV	0.680	0.782	0.803	0.818	0.833	0.855	0.958	0.818	0.022	0.277
	NPV	0.567	0.720	0.744	0.761	0.777	0.801	0.930	0.761	0.025	0.363
	AUROC	0.772	0.836	0.855	0.868	0.881	0.900	0.948	0.868	0.020	0.176
**Random Forest**	Accuracy	0.675	0.744	0.768	0.784	0.800	0.823	0.892	0.784	0.024	0.216
	F1	0.687	0.746	0.769	0.785	0.801	0.824	0.884	0.785	0.024	0.198
	Sensitivity	0.665	0.755	0.777	0.793	0.809	0.832	0.895	0.793	0.023	0.229
	Specificity	0.584	0.731	0.754	0.771	0.787	0.811	0.927	0.771	0.024	0.344
	PPV	0.676	0.773	0.795	0.810	0.826	0.848	0.948	0.810	0.023	0.271
	NPV	0.555	0.709	0.733	0.750	0.767	0.791	0.908	0.750	0.025	0.354
	AUROC	0.757	0.823	0.843	0.857	0.870	0.890	0.928	0.857	0.020	0.171
**Artificial Neural Network**	Accuracy	0.689	0.742	0.765	0.781	0.797	0.820	0.877	0.781	0.024	0.188
	F1	0.677	0.735	0.758	0.774	0.791	0.814	0.888	0.774	0.024	0.211
	Sensitivity	0.672	0.757	0.780	0.796	0.811	0.834	0.884	0.796	0.023	0.212
	Specificity	0.591	0.728	0.752	0.768	0.785	0.808	0.928	0.768	0.024	0.337
	PPV	0.659	0.771	0.793	0.808	0.824	0.846	0.940	0.808	0.023	0.281
	NPV	0.550	0.708	0.732	0.749	0.766	0.790	0.912	0.749	0.025	0.361
	AUROC	0.751	0.812	0.833	0.847	0.861	0.881	0.949	0.847	0.021	0.198
**Adaptive Boosting**	Accuracy	0.683	0.736	0.759	0.775	0.791	0.815	0.885	0.775	0.024	0.202
	F1	0.674	0.735	0.758	0.775	0.791	0.814	0.890	0.775	0.024	0.216
	Sensitivity	0.671	0.759	0.781	0.797	0.813	0.835	0.889	0.797	0.023	0.217
	Specificity	0.585	0.732	0.756	0.772	0.789	0.812	0.941	0.772	0.024	0.356
	PPV	0.676	0.780	0.802	0.817	0.832	0.853	0.951	0.817	0.022	0.274
	NPV	0.567	0.709	0.733	0.750	0.767	0.791	0.929	0.750	0.025	0.362
	AUROC	0.755	0.829	0.848	0.862	0.875	0.895	0.929	0.862	0.020	0.175

*Note*: Summary of model metrics for each of the four machine learning techniques (XGBoost, Random Forest, Artificial Neural Network, and Adaptive Boosting) based upon the derived distribution using analytic formulas described within the study.

Abbreviations: AUROC, area under the receiver operator characteristic curve; NPV, negative predictive value; PPV, positive predictive value.

Table 2a shows the bootstrapped distribution for model feature importance statistics for each covariate for the selected XGBoost model. Table 2b shows the ADD for model feature importance statistics for each covariate for the selected XGBoost model. Again the values between the bootstrap simulation distribution and the ADD are very similar across the minimum, 5th percentile, 25th percentile, median, 75th percentile, 95th percentile, maximum, mean, and range. The SEs for the model statistics and for the gain statistics were significantly less variable from the ADD.

**Table 2a:** For the XGBoost model, a summary of model gain statistics for each covariate in the model based on bootstrap simulation.

Covariates	Minimum	5th Percentile	25th Percentile	Median	75th Percentile	95th Percentile	Maximum	Mean	SD	Range
Angina	0.225	0.288	0.316	0.334	0.0353	0.383	0.456	0.335	0.029	0.231
Cholesterol	0.148	0.209	0.228	0.24	0.252	0.269	0.326	0.24	0.018	0.178
Maximum heart rate	0.081	0.114	0.129	0.139	0.15	0.165	0.201	0.139	0.015	0.12
Age	0.059	0.082	0.095	0.103	0.112	0.124	0.156	0.103	0.013	0.097
Resting blood pressure	0.027	0.051	0.061	0.069	0.076	0.087	0.109	0.069	0.011	0.082
Sex	0.026	0.038	0.044	0.049	0.054	0.062	0.082	0.049	0.007	0.056
Fasting blood sugar	0.007	0.029	0.037	0.043	0.05	0.063	0.142	0.044	0.011	0.135
RestingECG	0.003	0.012	0.017	0.02	0.024	0.029	0.043	0.02	0.005	0.04

Abbreviation: ECG, electrocardiogram.

**Table 2b:** For the XGBoost model, a summary of model gain statistics for each covariate in the model based on analytical formulas described within this study.

Covariates gain statistic	Minimum	5th Percentile	25th Percentile	Median	75th Percentile	95th Percentile	Maximum	Mean	SD	Range
Angina	0.225	0.290	0.317	0.335	0.353	0.380	0.456	0.335	0.027	0.231
Cholesterol	0.148	0.199	0.223	0.240	0.257	0.281	0.326	0.24	0.025	0.178
Maximum heart rate	0.081	0.106	0.126	0.139	0.152	0.172	0.201	0.139	0.020	0.12
Age	0.059	0.074	0.091	0.103	0.115	0.132	0.156	0.103	0.018	0.097
Resting blood pressure	0.027	0.045	0.059	0.069	0.079	0.093	0.109	0.069	0.015	0.082
Sex	0.026	0.028	0.041	0.049	0.057	0.070	0.082	0.049	0.012	0.056
Fasting blood sugar	0.007	0.025	0.036	0.044	0.052	0.063	0.142	0.044	0.012	0.135
RestingECG	0.003	0.007	0.015	0.020	0.025	0.033	0.043	0.02	0.008	0.04

Abbreviation: ECG, electrocardiogram.

The Anderson–Darling test was completed to validate whether the point estimate for the mean and SD are sufficient to approximate the full model distribution are reported in Table 3. The bootstrap distribution for the model metrics and the feature gain statistics were not significantly different than a normal distribution.

**Table 3:** For the XGBoost models.

A		B	
Model metrics	Anderson–Darling *p*	Gain statistics	Anderson–Darling *p*
Balanced accuracy	0.53	Angina	0.23
Accuracy	0.44	Cholesterol	0.46
F1	0.46	Maximum heart rate	0.3
Sensitivity	0.18	Age	0.27
Specificity	0.36	Resting blood pressure	0.7
PPV	0.22	Sex	0.18
NPV	0.97	Fasting blood sugar	0.99
AUROC	0.64	RestingECG	0.1

*Note*: (A) Summary of Anderson–Darling test for normality for model metrics. (B) Summary of Anderson–Darling test for normality for gain statistics for model covariates.

Abbreviations: AUROC, area under the receiver operator characteristic curve; NPV, negative predictive value; PPV, positive predictive value; RestingECG, resting electrocardiogram.

**Figure 1:**
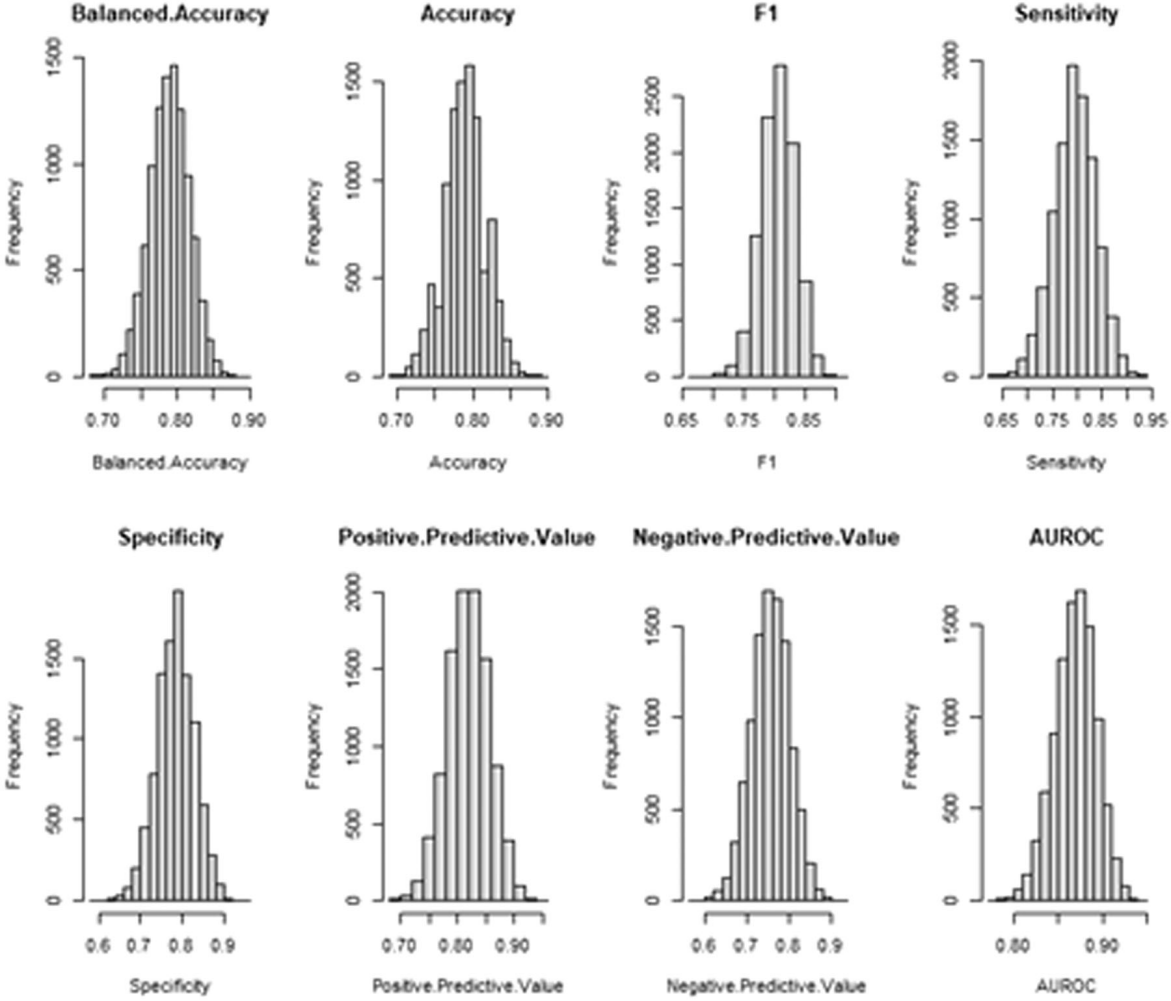
Balanced accuracy, Accuracy, F1, Sensitivity, Specificity, Positive predictive value, Negative predictive value, and area under the receiver operator characteristic curve (AUROC) for the XGboost model following bootstrap simulation.

Figure 1 shows the bootstrapped values for the model metrics (Balanced accuracy, Accuracy, F1, Sensitivity, Specificity, PPV, NPV, AUROC) for the XGBoost model.

**Figure 2:**
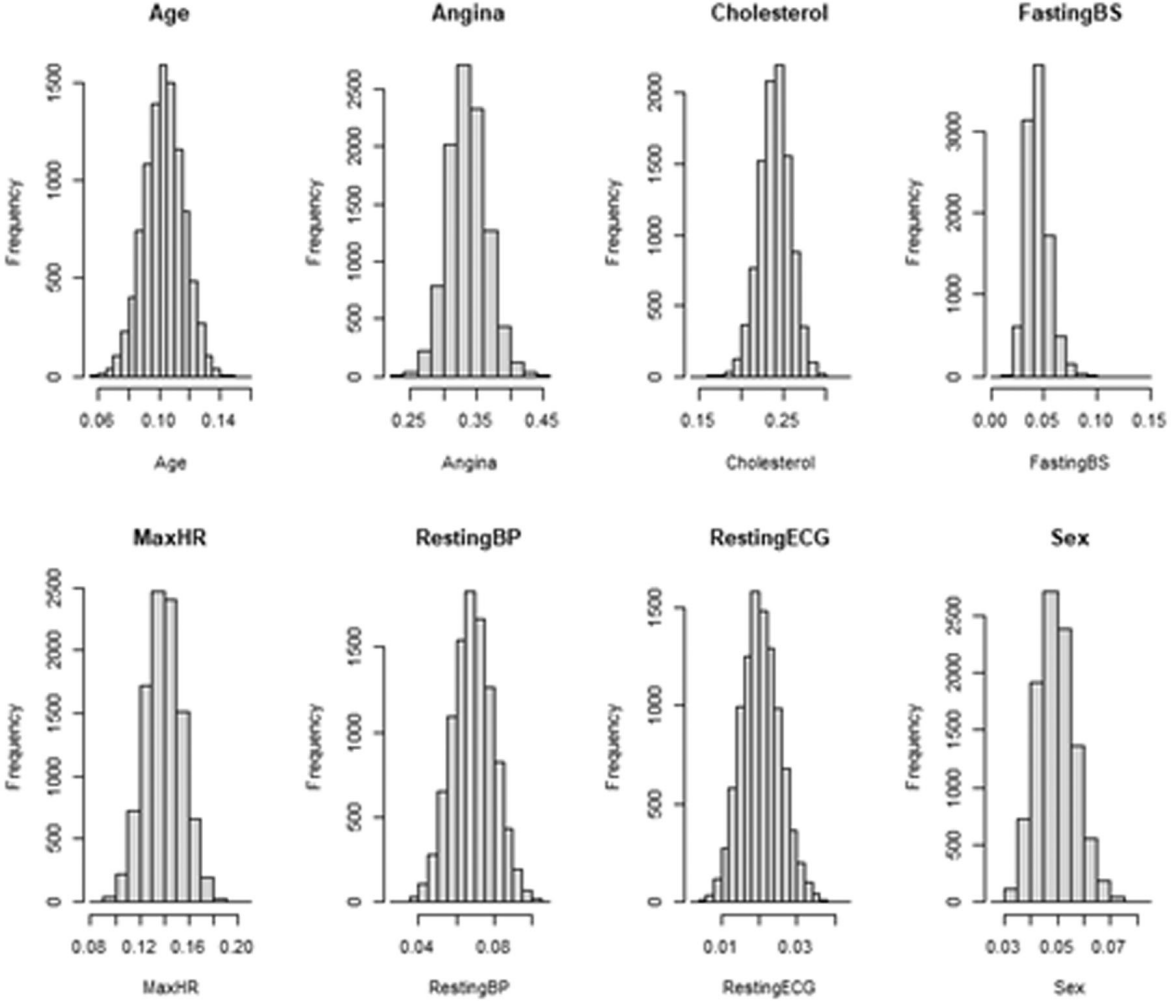
For the XGBoost models, the distribution of the gain statistic for all covariates: Age, Angina, Cholesterol, Fasting blood sugar (Fasting BS), Maximum heart rate (MaxHR), Resting blood pressure (RestingBP), Resting electrocardiogram (RestingECG), and Sex.

Figure 2 shows the bootstrapped distribution of gain statistics calculated for covariates that included Age, Angina, Cholesterol, Fasting blood sugar, Maximum heart rate, Resting blood pressure, RestingECG, and Sex.

Figures 1 and 2 validates the observations of the Anderson–Darling test demonstrating no significant difference between the bootstrapped distribution of the model metrics and feature gain statistics from a normal distribution.

## 4 DISCUSSION

We observed that the model metrics and model feature importance statistics for machine learning models converged on a Gaussian distribution in this retrospective, cross‐sectional cohort of heart disease patients. ADDs were used to calculate sufficient Gaussian distribution statistics, including the mean and SD. It was found that there was no significant difference in the overall distribution between the Gaussian approximation of the distribution for model metrics and feature importance statistics.

Bootstrapping has previously been the primary method used to derive accuracy statistics for machine learning model distributions.^18^, ^20^, ^24^, ^25^ Bootstrapping can generate a distribution based on data without any knowledge of the distribution and without violating any assumptions that are required to utilize a distribution for inference.^16^, ^17^, ^26^, ^27^, ^28^, ^29^ As a result, the vast majority of packages focus upon bootstrapping and thus so too do the vast majority of studies.^1^, ^8^, ^30^, ^31^, ^32^ Due to the increased computational power, these nonparametric methods can be completed efficiently and are especially useful for distributions that cannot be quantified analytically.^1^, ^3^, ^5^, ^22^, ^33^, ^34^, ^35^ The fact that bootstrapping relies on simulation is its weakness, making it difficult to replicate in other studies. Furthermore, due to differences in simulation methodology, it may be difficult to compare the results of the simulation if the results of the simulation are not summarized identically.

If a researcher wants to replicate the distribution of a study, they cannot replicate a bootstrapped distribution; however, with sufficient statistics of a random value distribution, the distribution can be exactly generated.^36^ Thus, identification of whether the results of the simulation‐based methods can be approximated with well‐established random variable distributions and their sufficient statistics computed will effectively allow comparisons of models within and between studies.

What our study uniquely contributes to the medical and biostatistics literature is a rigorous comparison between bootstrap simulations and a distribution generated from a Gaussian distribution using the point‐estimate for the mean and SDs (ADD). The use of Anderson–Darling methodology to evaluate bootstrap distributions to test mortality and validate visual judgements of histograms for the normal distribution allow for strong evidence that regardless of the skewed accuracy results that come from potential imbalances of data sets and machine learning methods, the overall distribution of model metrics follow a Gaussian distribution.

The study's findings can be broadly applied to research on machine learning. To begin, they can be persuaded to employ a variety of machine‐learning techniques and choose the most effective one, rather than relying solely on a single point estimate. Instead, a thorough evaluation of the estimate variances for the model metrics can be used to accurately determine which model is the most effective. As a result, we advocate that the strongest model is not only the one with the highest AUROC point estimate on a randomly selected seed but also the one with the highest distribution of multiple model accuracy statistics.^16^, ^17^, ^24^, ^25^, ^37^, ^38^, ^39^ Furthermore, the results of this study support that the distribution of each model metric follows a normal distribution and can be modeled analytically through the Gaussian distribution and the Whitney–Mann distribution for the AUROC, which we have termed the ADD pronounced the “AD distribution.”

### 4.1 Limitations

This study has several strengths and weaknesses. The study utilizes data from onlyappone cohort and thus may be difficult to generalize to other populations. However, as the goal was to evaluate methods to compute the variance of machine learning model statistics instead of developing models for heart disease, this is less of a concern. In addition, this study's replicability is enhanced by making use of a publicly accessible data set that is already integrated into an R package, which is in line with the paper's general recommendations. In addition, to acquire a better comprehension of the distribution of the model metrics and feature importance statistics computed from machine learning methods, subsequent studies will need to validate this approach with additional cohorts, both smaller and larger in size.

## 5 CONCLUSION

The distribution of model metrics and feature importance measures can be summarized by making use of the Gaussian distribution and the adequate statistics of mean and SD. Based on point estimates for the model metrics, there is no significant difference between the bootstrap distribution and the Gaussian distribution. Further retrospective and prospective cohort studies utilizing the model is needed to verify the conclusion.

## AUTHOR CONTRIBUTIONS

**Alexander A. Huang**: Conceptualization; formal analysis; investigation; methodology; resources; software; supervision; visualization; writing—original draft; writing—review & editing. **Samuel Y. Huang**: Conceptualization; formal analysis; investigation; methodology; resources; validation; writing—original draft; writing—review & editing. All authors have read and approved the final version of the manuscript. Corresponding author had full access to all of the data in this study and takes complete responsibility for the integrity of the data and the accuracy of the data analysis.

## CONFLICT OF INTEREST STATEMENT

The authors declare no conflict of interest.

## TRANSPARENCY STATEMENT

The lead author Samuel Y. Huang affirms that this manuscript is an honest, accurate, and transparent account of the study being reported; that no important aspects of the study have been omitted; and that any discrepancies from the study as planned (and, if relevant, registered) have been explained.

## Data Availability

The data sets generated and analyzed within this study are available through the national health services R community at https://nhsrcommunity.com/ and through the MLDataR package.
